# Hemodynamic Abnormalities in the Aorta of Turner Syndrome Girls

**DOI:** 10.3389/fcvm.2021.670841

**Published:** 2021-06-01

**Authors:** Lauren Johnston, Ruth Allen, Pauline Hall Barrientos, Avril Mason, Asimina Kazakidi

**Affiliations:** ^1^Department of Biomedical Engineering, University of Strathclyde, Glasgow, United Kingdom; ^2^Department of Radiology, Royal Hospital for Children, Glasgow, United Kingdom; ^3^Department of Paediatric Endocrinology, Royal Hospital for Children, Queen Elizabeth University Hospital, Glasgow, United Kingdom

**Keywords:** Turner syndrome, computational fluid dynamics, hemodynamics, cardiovascular disease, pediatric medicine, patient-specific, disturbed flow, atherosclerosis

## Abstract

Congenital abnormalities in girls and women with Turner syndrome (TS), alongside an underlying predisposition to obesity and hypertension, contribute to an increased risk of cardiovascular disease and ultimately reduced life expectancy. We observe that children with TS present a greater variance in aortic arch morphology than their healthy counterparts, and hypothesize that their hemodynamics is also different. In this study, computational fluid dynamic (CFD) simulations were performed for four TS girls, and three age-matched healthy girls, using patient-specific inlet boundary conditions, obtained from phase-contrast MRI data. The visualization of multidirectional blood flow revealed an increase in vortical flow in the arch, supra-aortic vessels, and descending aorta, and a correlation between the presence of aortic abnormalities and disturbed flow. Compared to the relatively homogeneous pattern of time-averaged wall shear stress (TAWSS) on the healthy aortae, a highly heterogeneous distribution with elevated TAWSS values was observed in the TS geometries. Visualization of further shear stress parameters, such as oscillatory shear index (OSI), normalized relative residence time (RRTn), and transverse WSS (transWSS), revealed dissimilar heterogeneity in the oscillatory and multidirectional nature of the aortic flow. Taking into account the young age of our TS cohort (average age 13 ± 2 years) and their obesity level (75% were obese or overweight), which is believed to accelerate the initiation and progression of endothelial dysfunction, these findings may be an indication of atherosclerotic disease manifesting earlier in life in TS patients. Age, obesity and aortic morphology may, therefore, play a key role in assessing cardiovascular risk in TS children.

## Introduction

Turner syndrome (TS) is a rare genetic disorder where the second sex chromosome in females is partially or completely absent and can affect all or only a percentage of cells (mosaicism). Although rare, TS is the most common chromosomal abnormality among females, affecting 1 in 2,500 live births based on epidemiological data from Europe, East Asia, and North America (1–3). The clinical characteristics of TS are highly variable, however, with congenital heart abnormalities estimated to occur in as many as half of individuals (4). These defects predominantly affect the left side of the heart, with the most commonly reported being an elongated transverse aortic arch (ETA) (5, 6). Defined as an increased distance between the second (LCCA) and third (LSA) supra-aortic branch origins, ETA is reported in 49% of TS adults (5, 6). Bicuspid aortic valve (BAV) is the second most common abnormality in TS, affecting ~30%, closely followed by aortic dilatation with a prevalence of 27% (5, 7, 8). Coarctation of the aorta (CoA) is reported in around 16% of TS females, most commonly at the site between the third supra-aortic branch and the descending aorta (5, 7, 8). Aberrant right subclavian artery (RSA) is an anatomical variation of the RSA which atypically originates from the arch as a separate fourth branch and has a prevalence of 8% in TS (6). These congenital abnormalities, alongside an underlying predisposition to obesity and hypertension, contribute to a greater risk of cardiovascular disease and ultimately reduced life expectancy in TS (9).

Hemodynamic factors have been linked to the initiation and development of cardiovascular disease for over a century (10). However, the exact nature of pro-atherogenic flow is uncertain with researchers proposing contradicting theories (11, 12). Fry suggested that high wall shear stress (WSS) preceded endothelial dysfunction, one of the early biological markers for atherosclerotic lesions that underlie most cardiovascular diseases (11). Soon after, Caro et al. suggested that high WSS regions are in fact spared from disease, with low WSS areas prone to develop atherosclerosis (12, 13). The oscillatory shear index (OSI) was then put forward by Ku et al. (14) to characterize regions of reversing flow, and today the combined low and/or oscillatory WSS theory is generally accepted as the biological mechanism for atherosclerosis. However, atherosclerotic lesions appear to depend on and vary with age (15): studies in human fetuses, newborns, and children have demonstrated the development of sudanophilic lesions downstream of branch ostia in the thoracic aorta (16); in young adults, lesions are observed laterally of such branch origins, while in middle age, upstream (17, 18); last, in older people, atherosclerotic lesions develop around the origins of intercostal arteries (19). Some aspects of such distinct lesion distributions have been described before hemodynamically and were correlated with strikingly varied WSS patterns calculated numerically around aortic branches (20–22).

In recent decades, computational fluid dynamic (CFD) modeling has gradually replaced traditional experimental methods in the study of cardiovascular diseases, largely due to its ability to quantify variables not measurable *in-vivo*, particularly at a temporal and spatial resolution exceeding all other methodologies (23). Increasingly complex models of arterial vessels have improved our understanding of the relationship between anatomical and hemodynamic factors (24–31). However, the accuracy of these models is dependent on several assumptions. The assumption of blood as an incompressible, Newtonian fluid is common in the case of larger arteries. While this assumption is not physiologically correct, qualitative wall shear stress patterns have proven to be comparable between Newtonian and non-Newtonian simulations (32). Multiple studies ignore the pulsatile nature of blood flow, assuming instead a steady-state condition (25, 26). This assumption is computationally-efficient when only considering the mean WSS result, but pulsatility is crucial for capturing time-dependent parameters such as instantaneous velocities and WSS, time-averaged wall shear stress (TAWSS), transverse WSS (transWSS), oscillatory shear index (OSI), and relative residence time (RRT) (33). In addition to the above assumptions, the computational result is highly sensitive to the assigned boundary conditions (BCs) (32–36). Madhavan and Kemmerling (32) compared five different inlet velocity profiles on human aortae and found only small differences in the flow solution approximately two diameters downstream from the aortic inlet. In a similar study on mice, Van Doormaal et al. (37) used realistic MRI-derived aortic root velocity profiles and suggested that idealized inflow profiles should be avoided. Pirola et al. (28) recommended the use of a 3D inlet velocity profile for hemodynamic analysis of the ascending aorta and arch, but a 1D inlet velocity profile was acceptable for evaluating flow in the descending aorta. Outlet BCs impact a greater percentage of the solution domain, but often *in-vivo* data is not available, which is a hurdle in patient-specific simulations (32). In the absence of *in-vivo* data, most studies apply either an outflow boundary condition, in which a percentage of the total flow is specified at each outlet, or a simple downstream resistance or the Windkessel model, in which the resistance and capacitance of the downstream vasculature are modeled (38). However, implementation of the Windkessel model is particularly challenging for children, due to the lack of values for the Windkessel parameters in the literature, especially for children with TS. In the absence of patient information, Murray's Law or the splitting method, both established from the power law relationship between branch diameter and flow rate, could be used (39, 40). Several authors have favored Murray's law over the zero-pressure boundary condition, for capturing more physiologically relevant flow features (33, 39).

In Turner syndrome, deviations from an anatomically healthy aorta are common, and therefore changes in blood flow may exacerbate the risk of cardiovascular disease. Understanding the pathogenesis of the increased cardiovascular morbidity and mortality in Turner syndrome, and determining the contributions of atherosclerotic disease, hypertension, and obesity was made a key cardiovascular research priority by the American Heart Association (4). To this end, our research primarily aims at providing new evidence for the aortic arch hemodynamics in the TS children population, with categorically abnormal aortic morphologies. Comparison with three age- and gender-matched, anatomically healthy cases is also made to highlight the differences with normal anatomies. The hemodynamic environment of each patient was studied, and the relationship between aortic morphology and flow was analyzed. Moreover, this study provides further evidence on understanding the aortic flow development generally in children and attempts to find correlations between hemodynamics and clinical significance in TS patients.

## Materials and Methods

### Magnetic Resonance Imaging (MRI) and Patient Cohort

In this study, retrospective MRI scans were obtained from four (*n* = 4) girls (average age 13 ± 2 years) with karyotypically proven Turner syndrome (Table 1), attending the pediatric TS clinic at the Royal Hospital for Children, Queen Elizabeth University Hospital (RHC, QEUH). TS3 underwent anomalous pulmonary venous drainage repair ~6 years prior to MRI imaging, and TS4 underwent left congenital diaphragmatic hernia repair at birth (13 years before the MRI scan). Both surgical corrections were unrelated to the region of interest, being the proximal aorta.

**Table 1:** Biometric and anatomical data for the Turner syndrome (TS) girls (*n* = 4).

	**TS 1**	**TS 2**	**TS 3**	**TS 4**
Age, *y*	14	10	14	13
Height, cm	136.60	134.70	143.70	138.30
Weight, kg	47.85	44.15	60.20	33.00
BSA, m^2^	1.3	1.27	1.53	1.2
BMI	25.6	24.3	29.2	17.3
BMI percentile	92nd	97th	99th	27th
BMI category	over-weight	obese	obese	healthy
BP_syst_/BP_diast_, mmHg	116/75	102/61	104/43	111/69
CO, l/min	4.58	5.40	4.97	3.34
HR, bpm	88	105	84	83
Anatomical abnormality	dilatation	ETA	aberrant RSA	none

*Age, height, weight, and blood pressure (BP) obtained from the patient electronic clinical records, as well as body surface area (BSA) and body mass index (BMI). The cardiac output (CO) and heart rate (HR) was obtained from within Medviso Segment software (http://medviso.com/segment)*.

All cardiac imaging was performed between 2014 and 2018 on a 1.5 Tesla diagnostic MRI scanner (MAGNATOM Aera/Avanto, Siemens Healthcare, GmbH). The scans were acquired with both ECG and respiratory gating. Additional 2D time-resolved phase-contrast MRI (PC-MRI) data was acquired at the aortic root of each individual over a cardiac cycle, oriented axially and centered on the anatomical position of the pulmonary artery. Flow in the through-plane direction was measured with PC-MRI imaging using a velocity encoding (VENC) of 150–400 cm/s and TE = 2.66–3.33 ms, TR = 9.94 ms (slice thickness of 3.5–5 mm; FOV = 300 × 300 mm), for all data, resulting in a 256 x 256 pixel matrix and resolution of 1.17 × 1.17 × 3.5–5 mm.

Biometric data (height, weight and blood pressure, BP) was obtained within 1 month of the cardiac imaging data and body mass index (BMI) and BMI percentile were computed according to the 1990 British childhood standards (Table 1) (41). The cardiac output (CO) and heart rate (HR) were obtained from the PC-MRI data. Patient-specific volumetric image and geometrical data from three gender-matched (average age 13 ± 6.8 years) patients (*n* = 3), with no known aortic abnormalities, were purchased from the Vascular Model Repository (www.vascularmodel.com). The average CO and HR of the healthy group were 3.77 ± 1 l/min (mean ± SD), and 81.33 ± 22.59 bpm (mean ± SD), respectively. Even though detailed BMI information was not included as part of the purchased data, it is expected that all healthy cases were within the normal BMI range, in accordance with the standard of the repository.

### Anatomical Reconstruction and Mesh Generation

**Figure 1:**
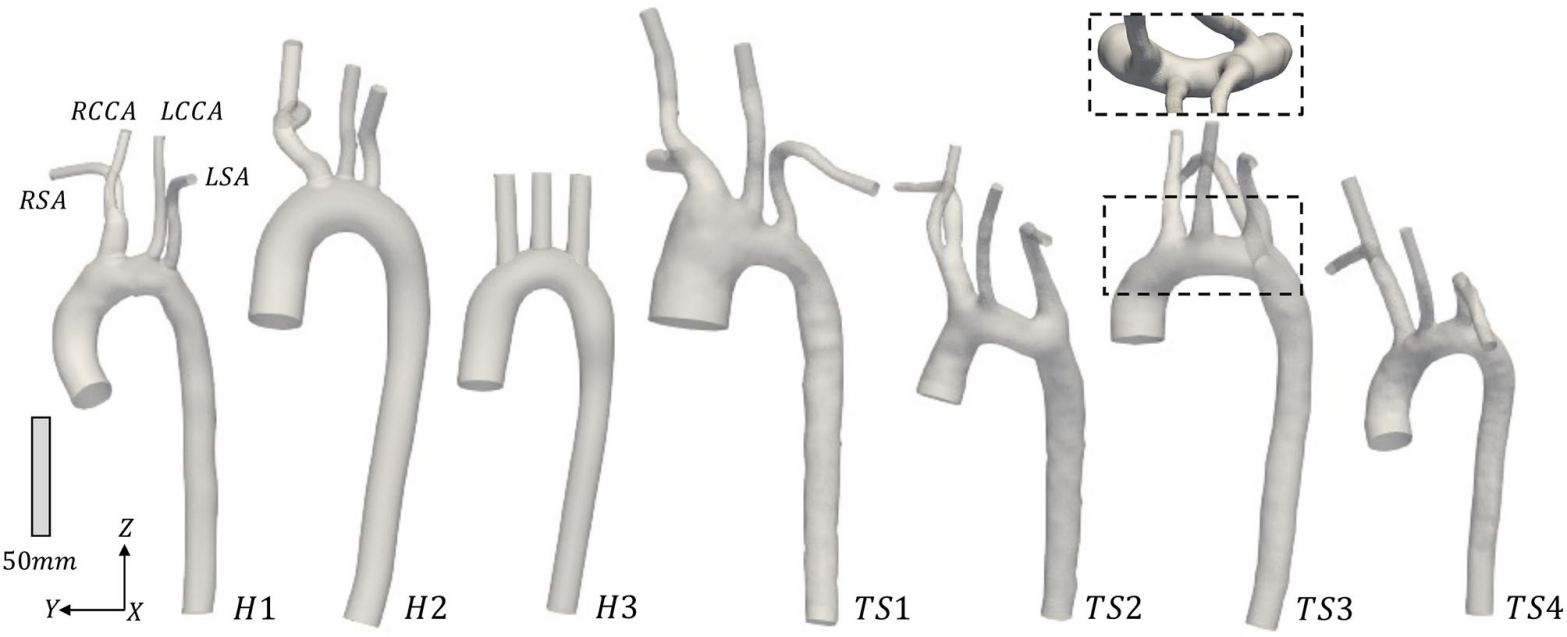
Anterior view of the reconstructed aortic models from the (H1-H3) healthy and (TS1-TS4) Turner syndrome (TS) girls. RSA, Right subclavian artery; RCCA, right common carotid artery; LCCA, left common carotid artery; LSA, left subclavian artery. Inset: Superior view of TS3 to highlight the origin of the aberrant RSA. Geometries are in scale.

The three-dimensional geometries of the TS group were segmented and reconstructed from the MRI data, from above the location of the aortic valve to the end of the thoracic aorta, including the brachiocephalic, left common carotid, and subclavian arteries, using the medical-imaging software ITK-SNAP (www.itksnap.org). In Figure 1 the anterior view of the reconstructed aortic models of the healthy (H1–H3) and Turner syndrome girls (TS1–TS4) is shown, with the geometries being in scale for direct comparison. The healthy controls had aortic diameters (*D*) of 17.53-22.48 mm at the sinotubular junction (Table 2), similar to the mean diameter of 17.5 mm (range = 11.1–26.4 mm) found in healthy children of a related age (*n* = 53, range = 2–20 years) (42). Ascending aorta diameters in women with TS are generally about 10% greater than in healthy women (43). In our cohort, the TS girls had aortic diameters of 18.99–33.77 mm (Table 2). The excessively large aortic diameter in TS1 was expected due to the dilated ascending aorta. Among the Turner syndrome models were aortic abnormalities commonly reported in the literature, such as ascending aorta dilatation (TS1), elongated transverse arch (TS2 and TS3), and an aberrant right subclavian artery (TS3), as stated in the patient clinical records. Arch morphology in TS4 was suggestive of a triangular-shaped “gothic” arch, although this was not recorded in the clinical records. Both TS1 and TS3 had a bicuspid aortic valve, while TS2 and TS4 had a normal functioning tricuspid aortic valve. TS girls 1, 2, and 4 exhibited the conventional anatomy of three supra-aortic vessels arising from the arch. In TS3, the right subclavian artery arose from the posterior arch, distal to the left common carotid artery (Figure 1, inset). There were no aortic abnormalities in the healthy cases (H1–H3).

**Table 2:** Hemodynamic information for healthy (H1–H3) and Turner syndrome (TS1–TS4) patients.

	**H1**	**H2**	**H3**	**TS 1**	**TS 2**	**TS 3**	**TS 4**
*D, mm*	17.53	22.48	19.68	33.77	19.30	22.56	18.99
*T, s*	0.57	1.00	0.76	0.68	0.57	0.71	0.72
$Q_{mean}\;\left[\frac{ml}{s}\right]$	48	82	56	77	90	83	56
$Q_{peak}\;\left[\frac{ml}{s}\right]$	197	302	223	254	294	320	207
*Re*_*mean*_	1,046	1,394	1,106	871	1,782	1,406	1,127
*Re*_*peak*_	4,292	5,145	4,336	2,873	5,820	5,418	4,164
*Wo*	16	15	16	28	18	18	15
Δ*y*_1_, *mm*	0.19	0.21	0.21	0.40	0.16	0.21	0.20
Δ*y*_*n* = 5_, *mm*	1.43	1.59	1.56	3.01	1.21	1.59	1.49

*T, cardiac cycle period; Q_mean_, Q_peak_: mean and peak flow rates, respectively, and Re_mean_, Re_peak_ the corresponding Reynolds numbers; Wo, Womersley number; Δy_1_, first boundary layer height; Δy_n = 5_, total boundary layer height (Equations 1–4)*.

**Figure 2:**
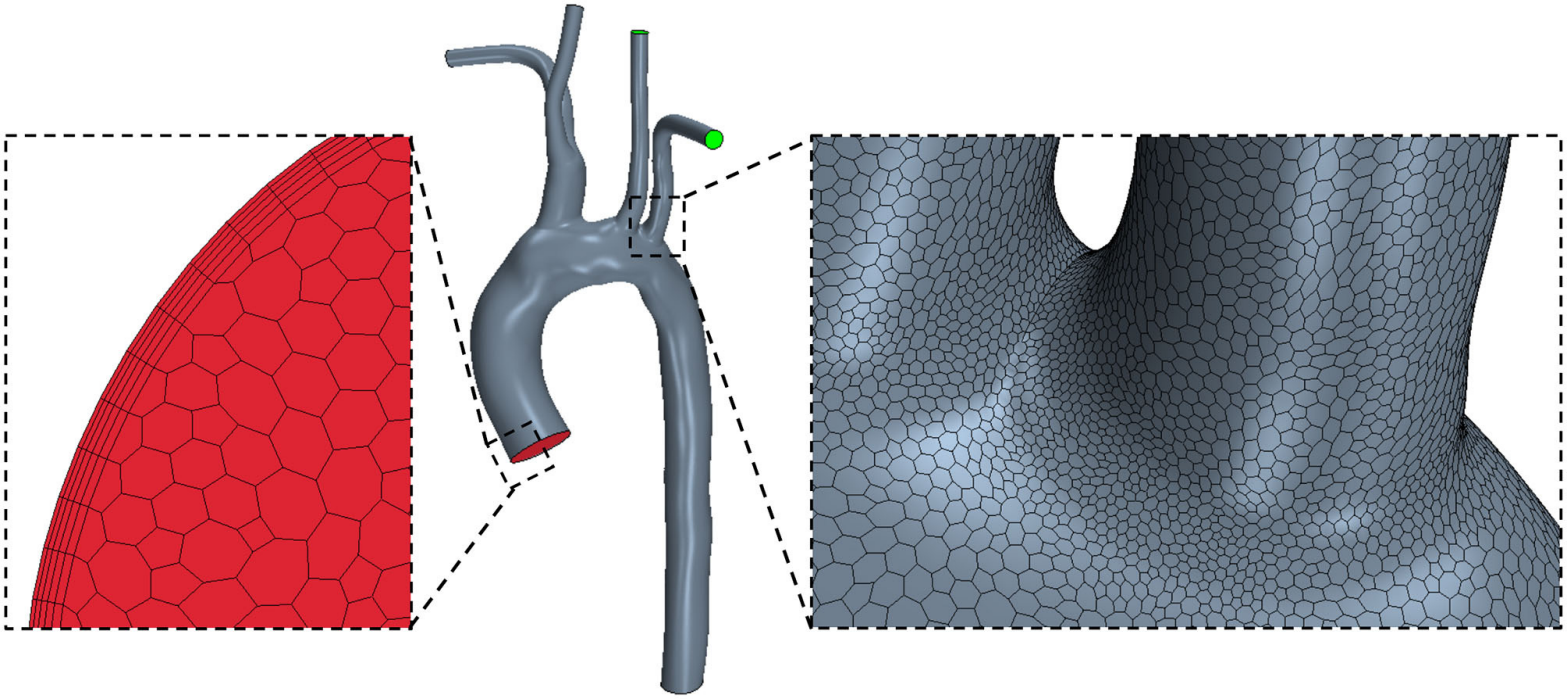
STAR-CCM+ polyhedral mesh shown on a healthy (H1) model with zoomed views of the inlet mesh with a prism boundary layer (left) and the arch surface mesh (right).

After segmentation, the surface models were smoothed in Autodesk Meshmixer (www.meshmixer.org) to reduce post-segmentation artifacts, and flow extensions were added normal to all boundary faces in VMTK (www.vmtk.org). An extension of half aortic diameter in length was added at each patient-specific inlet, which was fitted to a circular inlet of the same area. The domain was then discretized in STAR-CCM+ software (Siemens PLM, USA, www.plm.automation.siemens.com/global/en/products/simcenter/STAR-CCM.html) using polyhedral elements for the internal mesh, and prismatic elements for the boundary layer (Figure 2). While tetrahedral meshing is a more common approach, often a larger number of elements are required to limit element skewness and achieve acceptable mesh quality. Polyhedral meshing, introduced in STAR-CCM+, has two major benefits resulting in better numerical stability: one, each individual element has multiple neighbors, and two, the elements are less sensitive to stretching (44, 45). As a result, an accurate solution can be achieved with a much lower cell count of polyhedral elements than tetrahedral. Quantitative grid convergence was investigated on three meshes of increasing density (each grid was further refined by a factor of 2) and assessed using the Grid Convergence Index ($GCI=F_{s}\left(e/\left(r^{p}-1\right)\right)$ where *F*_*s*_ is a safety factor, taken as 1.25, *e* the relative error, *r* the mesh refinement ratio, and *p* the order of convergence) (46). The results confirmed that wall shear stress accuracy was suitably achieved with 1.5 million polyhedral elements (GCI ≅ 11%). Mesh generation with polyhedral elements is a promising discretization approach to reduce the computational time of simulations while retaining accuracy.

To accurately resolve the flow adjacent to the wall, the boundary layer mesh was generated using a total of 5 layers, with the height of the first layer (Δ*y*_1_) satisfying a y+ value of 1 (47), according to Equation (1). The y+ value describes a non-dimensional distance from the wall to the first element node and therefore characterizes near-wall mesh quality. Refining the near-wall mesh with an appropriate y+ value is crucial for resolving flow in the viscous sublayer of the boundary layer. The values of the first boundary layer height (Δ*y*_1_) and total boundary layer height (Δ*y*_*n* = 5_) are shown in Table 2.

(1) $$\begin{array}{l} \Delta y_{1}=\frac{\mu y+}{\rho \;U_{T}} \end{array}$$

where Δ*y*_1_ is the height of the first layer, *y*+ is equal to 1, μ and ρ are the fluid viscosity and density, taken as 3.5e-3 Pa s and 1050 kg/m^3^, respectively (25), and *U*_*T*_ is the friction velocity computed from Equation (2):

(2) $$\begin{array}{l} U_{T}=\sqrt{\frac{\tau_{w}}{\rho }} \end{array}$$

where τ_*w*_ is the wall shear stress, calculated according to Equation (3), *U*_*mean*_ is the mean velocity value corresponding to Q_mean_ (Table 2), and *C*_*f*_ is the skin friction coefficient calculated per Equation (4) (48).

(3) $$\begin{array}{l} \tau_{w}=\frac{1}{2}C_{f}\rho {U_{mean}}^{2} \end{array}$$

(4) $$\begin{array}{r} C_{f}=\;2\;*[{\left(\frac{8}{Re}\right)}^{12}\;+\;({\left(2.457\;\ln\left({\left(\frac{Re}{7}\right)}^{0.9}\right)\right)}^{16}+\\ {{{\left(\frac{37530}{Re}\right)}^{16})}^{-1.5}]}^{1/12} \end{array}$$

The adequacy of the y+ value was further confirmed from Equations (1, 2), based on the directly calculated maximum WSS values, which led to a y+ value much smaller than 1.

### Boundary Conditions

**Figure 3:**
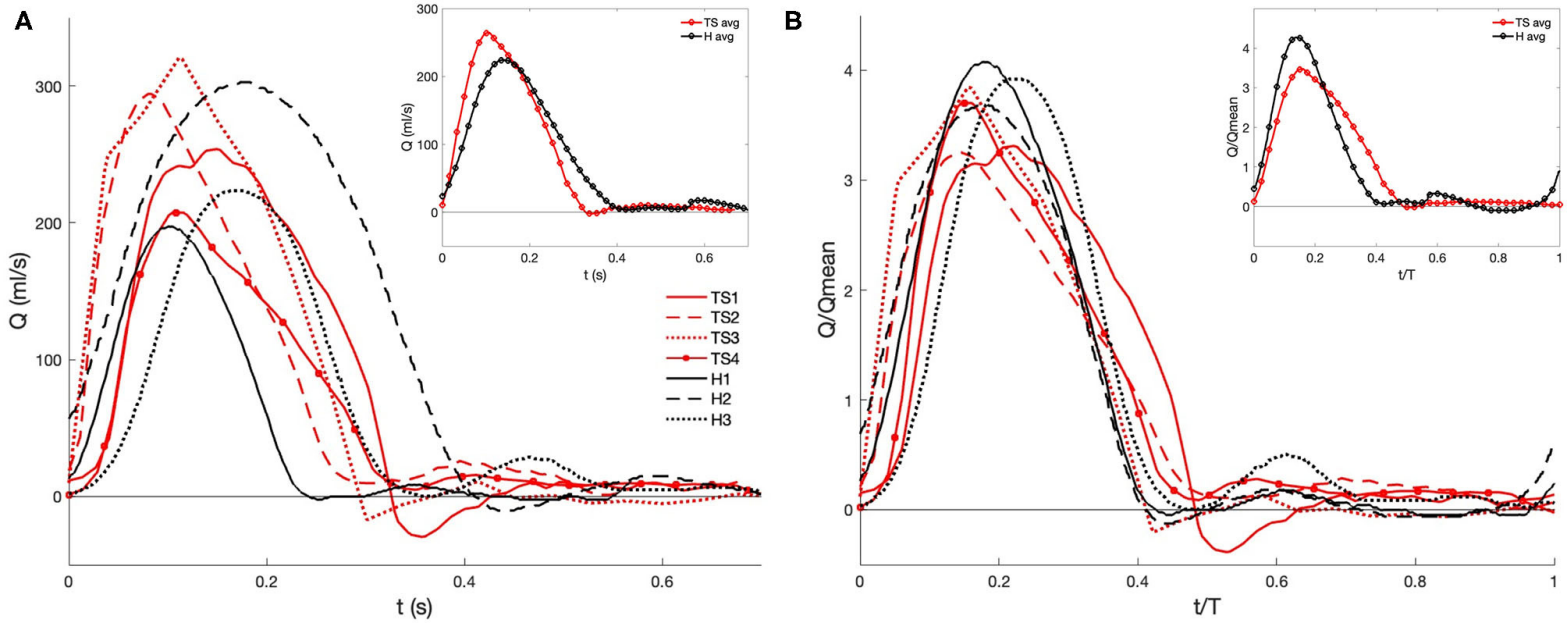
PC-MRI derived **(A)** volumetric flow rate and **(B)** normalized waveforms at the aortic root during one cardiac cycle for healthy (H) and Turner syndrome (TS) girls. Insets: average data calculated from H1-H3 (black line) and TS1-TS4 (red line). Flow rate and time normalized by the mean flow rate and cardiac cycle period (see Table 2 for values).

The flow exiting the aortic valve was segmented from the two-dimensional time-resolved PC-MRI data with the use of Medviso Segment software (http://medviso.com/segment), resulting in a series of time-dependent flow waveforms (Figure 3). The highest peak flow rate was found for TS2 and TS3, and the lowest peak flow rate for H1 (Figure 3A). To account for diameter and cardiac cycle variability, the normalized flow rates were calculated (Figure 3B), where two of the healthy girls demonstrated higher peak flow rates than the TS girls. The subject-specific volumetric flow waveforms (Figure 3A) were applied at the inlet boundary, which was defined at the location of the sinotubular junction.

The cardiac cycle period (T) and mean and peak flow rates (Q_mean_, Q_peak_, respectively) were extracted from the location of the aortic valve from the PC-MRI data, while the Reynolds and Womersley numbers were calculated post-segmentation using the patient-specific inlet diameter (*D*) and the cardiac cycle period (Table 2). The mean and peak Reynolds number (Re = 4Qρ/(πDμ) in TS patients varied, although the mean value for the TS group (Re_mean_=1296) was greater than the healthy group (Re_mean_ = 1,182). Available Re_peak_ data in the literature for TS children reported a range of 3,980–6,560 in the ascending aorta, meaning that the average value for our cohort (Re_mean_ = 4,568) was at the lower end of this range (49). The Womersley number is a non-dimensional expression of the pulsatile nature of blood flow, and was calculated based on Equation (5):

(5) $$\begin{array}{l} Wo=\frac{D}{2}\;\sqrt{\frac{2\;\pi \;f\;\rho }{\mu }} \end{array}$$

where *f* is the frequency of the cardiac cycle (*s*^−1^). The expected Womersley number in the abdominal aorta of a young healthy adult is 13 (50). At large Womersley numbers (above 10), the shape of the velocity profile is relatively flat or plug-like, with the maximum velocity no longer at the center (51). The computed Womersley number (Table 2) for the healthy aortae (Wo = 15–16) is within the range of TS2-TS4 (Wo = 15–18). For TS1, with the largest aortic diameter, the Womersley number is the highest with a value of 28.

The percentage of the total flow rate distributed to each outlet was calculated using Murray's law due to the shortage of patient-specific or literature values for children. In arterial bifurcations, Murray's law states that the flow is proportional to the diameter of that vessel raised to a power, *n* (39). For the right subclavian branch, it can be expressed as:

(6) $$\begin{array}{r} \frac{Q_{RSA}}{Q_{RSA}\;+\;Q_{RCCA}+Q_{LCCA}+Q_{LSA}+Q_{DescAO}}\;=\\ \frac{{D_{RSA}}^{n}}{{D_{RSA}}^{n}\;+\;{D_{RCCA}}^{n}+{D_{LCCA}}^{n}+{D_{LSA}}^{n}+{D_{DescAO}}^{n}} \end{array}$$

where *Q*_*RSA*_, *Q*_*RCCA*_, *Q*_*LCCA*_, *Q*_*LSA*_, *Q*_*DescAo*_ are the flow rates, and *D*_*RSA*_, *D*_*RCCA*_, *D*_*LCCA*_, *D*_*LSA*_, *D*_*DescAo*_ the diameters at the corresponding vessels. This relationship has been used in several human CFD studies, and is well-known with an exponential power of 3, hence the name “Murray's cube law.” However, several authors have shown that a power of 2 is more valid in the case of larger vessels such as the aorta (52–54). Using a power of 2, the calculated combined flow percentage to the aortic branches was 34–59% for the healthy aortae and 32–55% for the TS aortae, with the remaining flow distributed to the descending aorta (Table 3).

**Table 3:** Outlet flow percentages for each individual case calculated using Murray's Law [exponential power of 2, Equation (6)].

	**H1**	**H2**	**H3**	**TS 1**	**TS 2**	**TS 3**	**TS 4**
*Q*_*RSA*_	9%	14%	19%	20%	6%	8%	10%
*Q*_*RCCA*_	9%	14%		14%	9%	12%	17%
*Q*_*LCCA*_	7%	9%	19%	13%	9%	12%	10%
*Q*_*LSA*_	9%	12%	21%	8%	8%	5%	15%
*Q*_*DescAo*_	66%	51%	41%	45%	68%	63%	48%

In line with other CFD studies in the aortic arch, the arterial wall was presumed non-deformable and the no-slip BC was assigned (24, 25, 27, 28, 30, 31).

### Numerical Method

Throughout this study, the fluid was considered incompressible and Newtonian, with a constant density and viscosity. The fluid was governed by the time-dependent Navier-Stokes equations:

(7) $$\begin{array}{l} \nabla \cdot \vec{u}=0 \end{array}$$

(8) $$\begin{array}{l} \rho \frac{\partial u}{\partial t}+\rho \left(\vec{u}\cdot \nabla \right)\vec{u}=-\nabla p\;+\mu \nabla^{2}\vec{u} \end{array}$$

where $\vec{u}$ is the velocity vector and p the pressure.

Flow simulations were performed in the open-source software, OpenFOAM^©^ (www.openfoam.org, version 6), using the combined pressure-implicit split-operator and semi-implicit method for pressure-linked equations (PIMPLE) solver for incompressible, transient flow. The flow was computed using the wall-adapted local eddy-viscosity (WALE) large eddy simulation (LES) model, with temporal and spatial discretization performed using second order accurate schemes (backward Euler and central differencing, respectively). As the peak Reynolds numbers (Table 2) are in the transitional to turbulent range, an investigation was made with a pulsatile laminar model, a k-omega SST model, and the LES (WALE) model for the same mesh (TS2). The wall shear stress results were qualitatively similar for all three models, and marginally different quantitatively (0.14% difference in the integral of the WSS between the LES and laminar models, and 6% difference between the LES and k-omega SST models). However, the LES model more accurately captured the flow features, especially during systole and early diastole. The motivation for the use of the LES turbulence model was further reinforced by other published studies on blood flow (55, 56). Blood flow was simulated until time-periodicity was reached at approximately five cardiac cycles with a time step (*t*) of 1 × 10^−3^ s (satisfying mean Courant number < 1). Residual control for the convergence criteria was set to 1 × 10^−5^ (57) for both pressure and velocity.

### Shear Stress Parameters

The characterization of shear stress during pulsatile flow is commonly described with hemodynamic parameters such as the instantaneous wall shear stress (WSS) at various time points in the cardiac cycle, the time-averaged WSS (TAWSS), the oscillatory shear index (OSI), the relative residence time (RRT) and the transverse WSS (transWSS) (Equations 9–12, respectively). Such shear stress parameters can be used to identify areas where flow departs from a laminar, unidirectional pattern.

(9) $$\begin{array}{l} TAWSS=\frac{1}{T}\int_{0}^{T}|\vec{\tau_{w}}|\;dt \end{array}$$

(10) $$\begin{array}{l} OSI=\frac{1}{2}\left(1-\frac{\left|\frac{1}{T}\int_{0}^{T}\;\vec{\tau_{w}}\;dt\right|}{\frac{1}{T}\int_{0}^{T}|\vec{\tau_{w}}|\;dt}\right) \end{array}$$

(11) $$\begin{array}{l} RRT={\left[\left(1-2\;OSI\right)TAWSS\right]}^{-1} \end{array}$$

(12) $$\begin{array}{l} transWSS=\;\frac{1}{T}\int_{0}^{T}\left|\vec{\tau_{w}}\;.\;\left(\vec{n}\;x\;\frac{\int_{0}^{T}\vec{\tau_{w}}\;dt}{\left|\int_{0}^{T}\vec{\tau_{w}}\;dt\right|}\right)\right|\;dt \end{array}$$

where $\left|\vec{\tau_{w}}\right|$ is the magnitude of the wall shear stress vector, and $\vec{n}$ is the surface normal In this study, the TAWSS, OSI, RRT and transWSS were taken over the fifth cardiac cycle, and both the WSS and TAWSS were normalized with respect to the mean WSS at the inlet for each individual case. The OSI describes the degree of oscillatory flow ranging from zero, representing unidirectional flow, to 0.5, representing reversing flow with no mean shear direction (58). The RRT provides information on the residence time of flow particles in close proximity to the wall and is elevated in regions of low magnitude and high oscillatory WSS (59). RRT was normalized with respect to the surface integral average for the patient-specific aortic geometries. The transWSS quantifies multidirectional flow, with low values indicating that flow remains approximately parallel to a single axis throughout the cardiac cycle and high values indicating large changes in flow direction (60).

## Results

### Blood Flow in the Aorta

**Figure 4:**
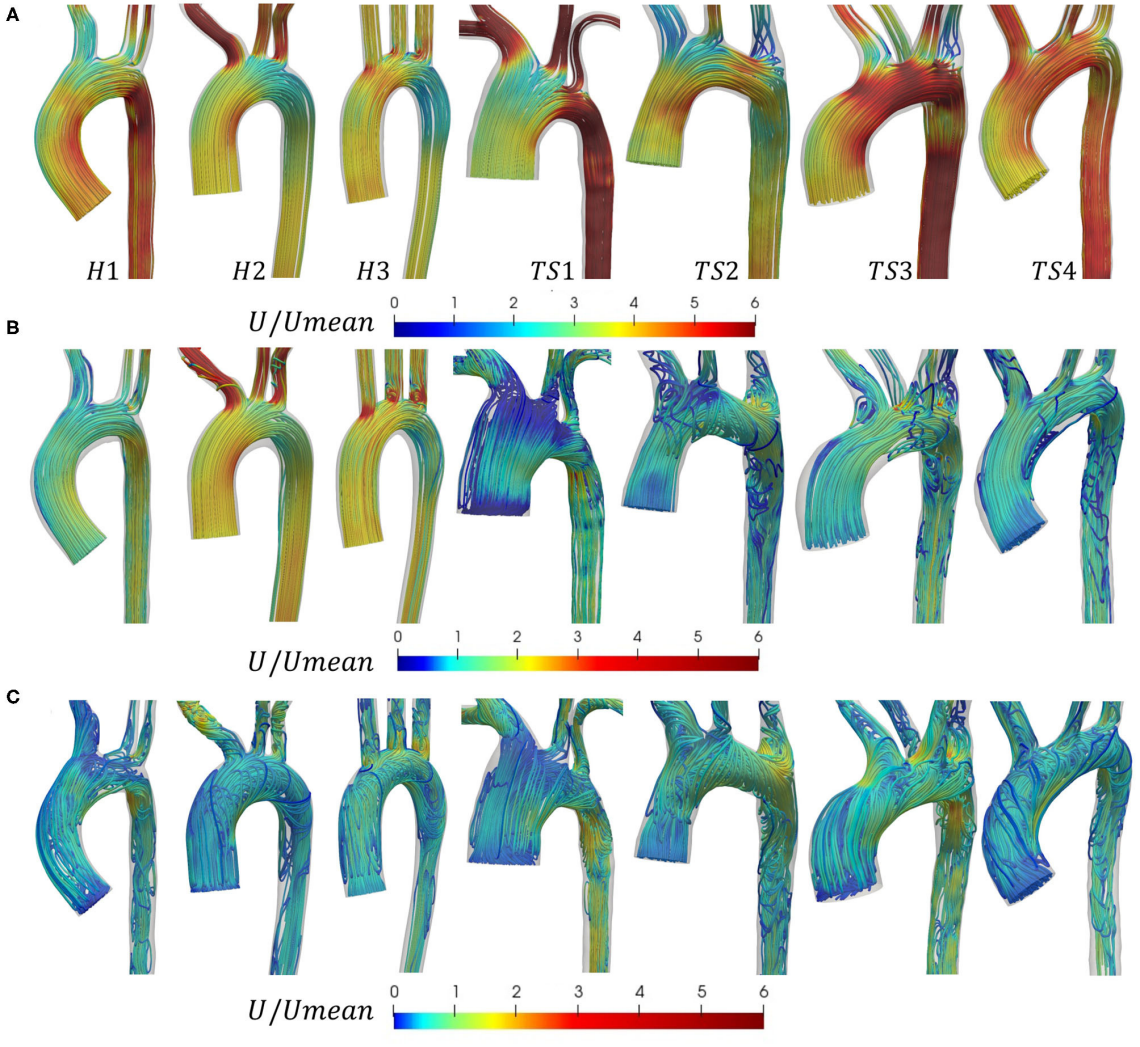
Velocity streamlines in the aortic arch of the healthy (H1-H3) and Turner syndrome (TS1-TS4) girls at **(A)** peak velocity (*t*_1_), **(B)** maximum deceleration (*t*_2_), and **(C)** mid-diastole (*t*_3_), colored by non-dimensional velocity magnitude that is normalized according to the average inlet velocity (*U*_*mean*_), derived from patient PC-MRI data (anterior view). Note that the color legends in **(B)** and **(C)** were shifted compared to **(A)** to enhance visualization. For interpretation of the colored legends, please refer to the online version of the paper.

**Figure 5:**
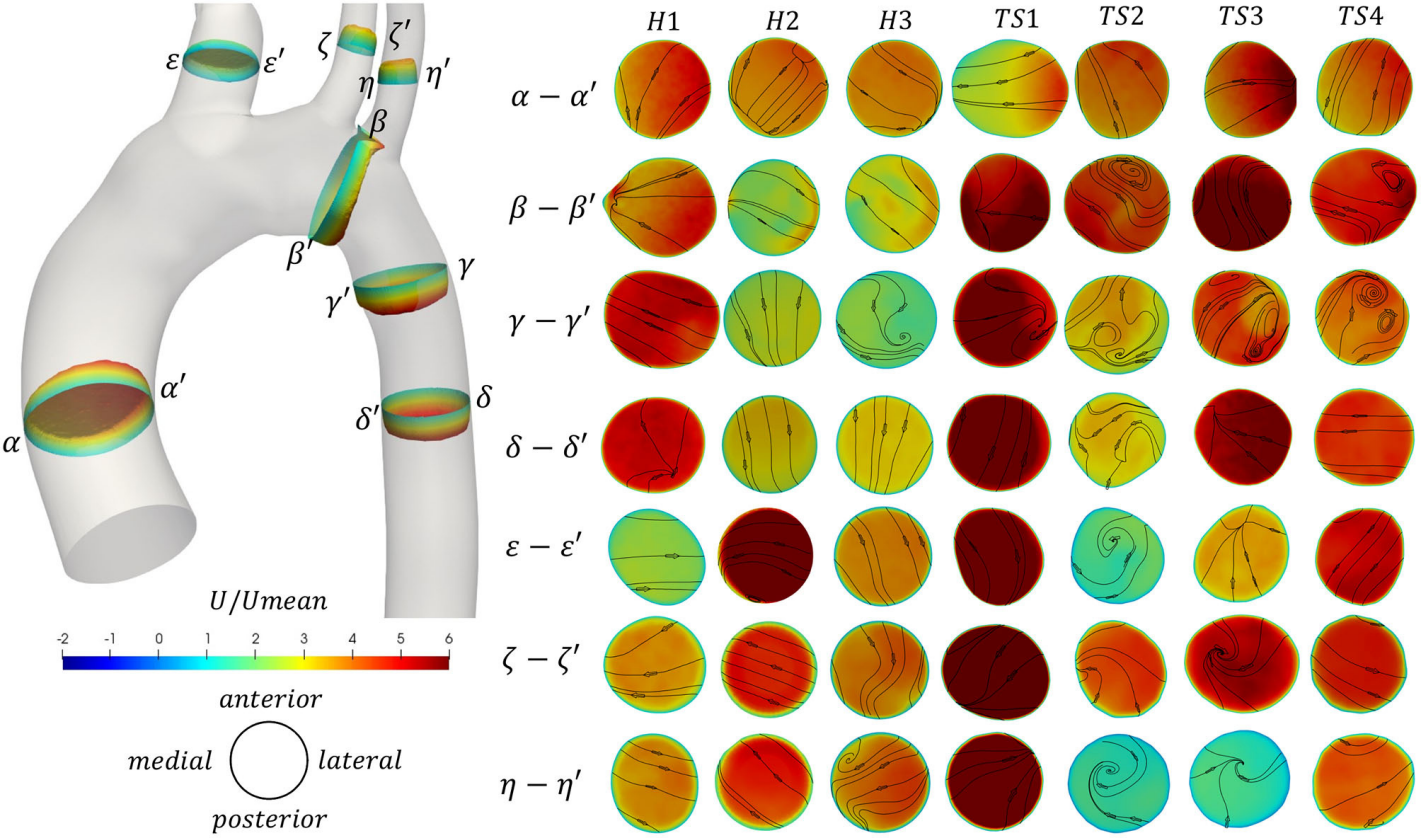
(Left) Through-plane velocity profiles and (Right) contours of through-plane velocity overlayed by vectors of in-plane velocity on seven cross-sections α-α′ to η-η′ along the aorta [locations shown on the 3D healthy (H1) model]. Contours colored by non-dimensional axial velocity at peak velocity for the healthy (H1–H3) and Turner syndrome (TS1–TS4) girls. Cross-sections are oriented looking downstream, with the top and bottom edges corresponding to the anterior and posterior sides of the aorta, respectively, and the left and right points as shown on the left. Cross-sections are not to scale.

Blood flow was visualized in the geometries of Figure 1, using a combination of 3D streamlines (Figure 4) at three time points in the cardiac cycle, peak velocity (*t*_1_), maximum deceleration (*t*_2_), and mid-diastole (*t*_3_), and 2D and 3D vector-fields (Figure 5) at peak velocity (*t*_1_). Maximum deceleration was defined as the minimum rate of change of the velocity with respect to time.

#### Velocity Streamlines

In Figure 4A, the non-dimensional velocity streamlines in the aortic arch were visualized at peak velocity (*t*_1_). In all three healthy cases (H1-H3), the flow at peak systole was laminar throughout the aortic arch, the descending aorta, and the three major branches arising from the arch. A similar flow pattern was observed for TS patients 1 and 4. In TS2 and TS3, the streamlines at peak systole were, for the most part, laminar throughout the aorta, except at the entrance to the LSA in both geometries, and the aberrant RSA in TS3, where small zones of recirculation were formed. In the ascending aorta of all cases, the velocity magnitude was greater at the inner wall. The majority of flow within the arch of H1–H2, and to some extent in H3 and TS2, was of lower magnitude than in the ascending aorta. In all TS aortae, the velocity magnitude for the majority of the arch was similar to, or greater than, the flow in the ascending aorta. In the majority of TS aortic models, flow velocity in the descending aorta was high, with the exception of TS2.

In Figure 4B, the non-dimensional velocity streamlines in the aortic arch were visualized at maximum deceleration (*t*_2_). In the healthy aortae, flow was relatively laminar with the exception of the entrance to the LCCA and LSA branches in H3. In contrast, a complex flow pattern with significant secondary flows was observed for all TS aortic models. In the ascending aorta of TS girls 2–4, the flow at maximum deceleration was laminar. However, in the TS girl with ascending aortic dilatation (TS1), the flow was highly disturbed in this region with significantly low velocity values. In the aortic arch of TS3–TS4, vortical flow developed along the lesser curvature and extended to the entrance of the descending aorta, while in TS1 and TS2 the region of vortical flow filled the majority of the aortic arch. In the descending aorta of the TS girls, with the exception of TS1, vortical flow was present at the proximal wall with undisturbed streamlines at the distal wall, with the inverse true for TS1. Unlike in the healthy geometries, the pattern of flow entering the TS aortic branches was unpredictable and highly disturbed. Strong vortical flow patterns were observed in the brachiocephalic branch of TS1, and recirculation regions at the proximal wall of TS2–TS4. The left common carotid artery of TS1 and TS3, and the left subclavian branch of TS1–TS3 further exhibit disturbed flow.

In Figure 4C, the non-dimensional velocity streamlines in the aortic arch were visualized at mid-diastole (*t*_3_). For the healthy aortae, the predominantly laminar flow pattern seen at maximum deceleration (Figure 4B) was replaced with strong, slow-moving vortical motion throughout the entire vessel at mid-diastole (Figure 4C). For TS1-TS2, there was little visible difference in the flow patterns between *t*_2_ (Figure 4B) and *t*_3_ (Figure 4C), albeit with lower velocity values and a slight increase in vortical flow in the ascending aorta of TS2 and descending aorta of TS1. For TS3, flow throughout all regions of the aorta and aortic branches was clearly more disturbed in mid-diastole than in systole. Similar observations could be made for TS4, with the greatest flow disturbance seen in the ascending aorta and arch.

#### Through-Plane and In-Plane Velocities

Furthermore, the through-plane and in-plane velocities were calculated for seven cross-sectional slices (α-α′ to η-η′, Figure 5) of all models, at peak velocity (*t*_1_). The 3D through-plane velocity profiles were shown only for a healthy (H1) case, in the corresponding geometry (left side of Figure 5), while the cross-sections, colored by through-plane velocity contours and overlaid by in-plane velocity streamlines, were shown to the right side of Figure 5 for all models. The cross-sectional slices were considered at the same relative locations for all cases, perpendicular to the aortic centerline and relative to the individual model inlet diameter, D: slice α-α′ was taken 1D upstream from the inlet; slice β-β′ was assumed midway between the LCCA and LSA branches; γ-γ′, 1D downstream from the LSA (TS3: from the LCCA); δ-δ′, 2D downstream from the LSA (TS3: from the LCCA); ε-ε′, 0.5D upstream from the brachiocephalic junction; ζ-ζ′, 0.5D upstream from the LCCA origin; and η-η′, 0.5D upstream from the LSA ostium. The slices in Figure 5 are oriented so that the top and bottom edges correspond to the anterior and posterior sides of the aorta, respectively, while the left and right points correspond to the greater and lesser curvature of the arch, respectively, for slices α-α′ to δ-δ′, and to the outer and inner walls of the branches, for slices ε-ε′ to η-η′. That is, all cross-sections are oriented looking downstream. In all figures, the velocity values were normalized according to the corresponding mean inlet velocity magnitude.

As the velocity increases to a maximum during peak systole (Figure 5), blood flow accelerates along the curvature of the arch, with a tendency to skew toward the inner wall of the ascending aorta (α′) as seen in TS1 and TS3, and to a lesser extent in H1 and TS4. In H2 and TS2 the flow was skewed anterolaterally, and in H3 the flow was uniform. In slice β-β′, the flow was skewed laterally or posterolaterally for all healthy aortae, posteriorly for TS1 and TS3, laterally for TS4, and posteromedially for TS2. At the entrance to the descending aorta, flow in the healthy aortae was only slightly skewed: toward the outer curvature wall (γ) in H1 and H3, and toward the inner curvature wall (γ′) in H2. In the TS aortae, flow was more visible skewed, specifically toward the outer curvature wall in TS1 and TS3, and the anterior wall in TS2. In the remainder of the descending aorta (δ-δ′), the flow field was well-structured with little asymmetry, except in TS2 where a region of higher velocity flow was located near the anteromedial wall. For slices ε-ε′ to η-η′, the individual branch anatomy of each aorta influenced the axial and transverse flow fields. When asymmetry of the axial flow was present, it tended to be skewed toward a region of the lateral wall, as seen in the brachiocephalic branch (ε-ε′) of TS2 and TS4, the left common carotid branch (ζ-ζ′) of H3 and TS2, and the left subclavian branch (η-η′) of H1. In the left common carotid branch (ζ-ζ′) of TS3, the flow velocity was greatest posterior to the vessel center.

Figure 5 also revealed further information on the presence of secondary flows at peak velocity that were not visible in Figure 4A. In the ascending aorta (α-α′), even though the flow was laminar for all cases presented (Figure 4A), the direction of secondary flow differed among the patients, although it was never in the direction of the posterior wall. By the time the flow reached the distal end of the aortic arch (β-β′), the secondary flow direction changed for all geometries, except for H3 and TS1, while recirculatory flow was present along the anterior, lateral, or anterolateral walls in TS2–TS4, respectively. Secondary flow in the first descending aortic slice (γ-γ′) of the healthy aortae was present as a single recirculation zone at the posterolateral wall of H3 exclusively. The same region of recirculating flow was present in the first descending aortic slice of TS1. In TS2–TS4, secondary flow in slice γ-γ′ presented as a pair of counter-rotating vortices at the posterior wall of TS2, the posterolateral wall of TS3, and the anterolateral wall of TS4. An additional region of recirculating flow was present at the anterior wall of TS3. In slice δ-δ′, taken further downstream in the descending aorta, secondary flow direction changed from slice γ-γ′ in all geometries except for H2, but remained laminar in all. In the majority of healthy aortic branches, excluding the BCA (ε-ε′) and LSA (η-η′) in H2 and H3 respectively, in-plane velocities were laminar. The same was true for all aortic branches of TS1 and TS4. In TS2, flow recirculation was present in the brachiocephalic branch (ε-ε′) near the anterior wall, and in the left subclavian branch (η-η′) near the anteromedial wall. A single recirculation region was present between the vessel center and medial wall in the LCCA branch (ζ-ζ′), and between the vessel center and anterior wall in the LSA branch (η-η′) of TS3.

### Other Hemodynamic Metrics

**Figure 6:**
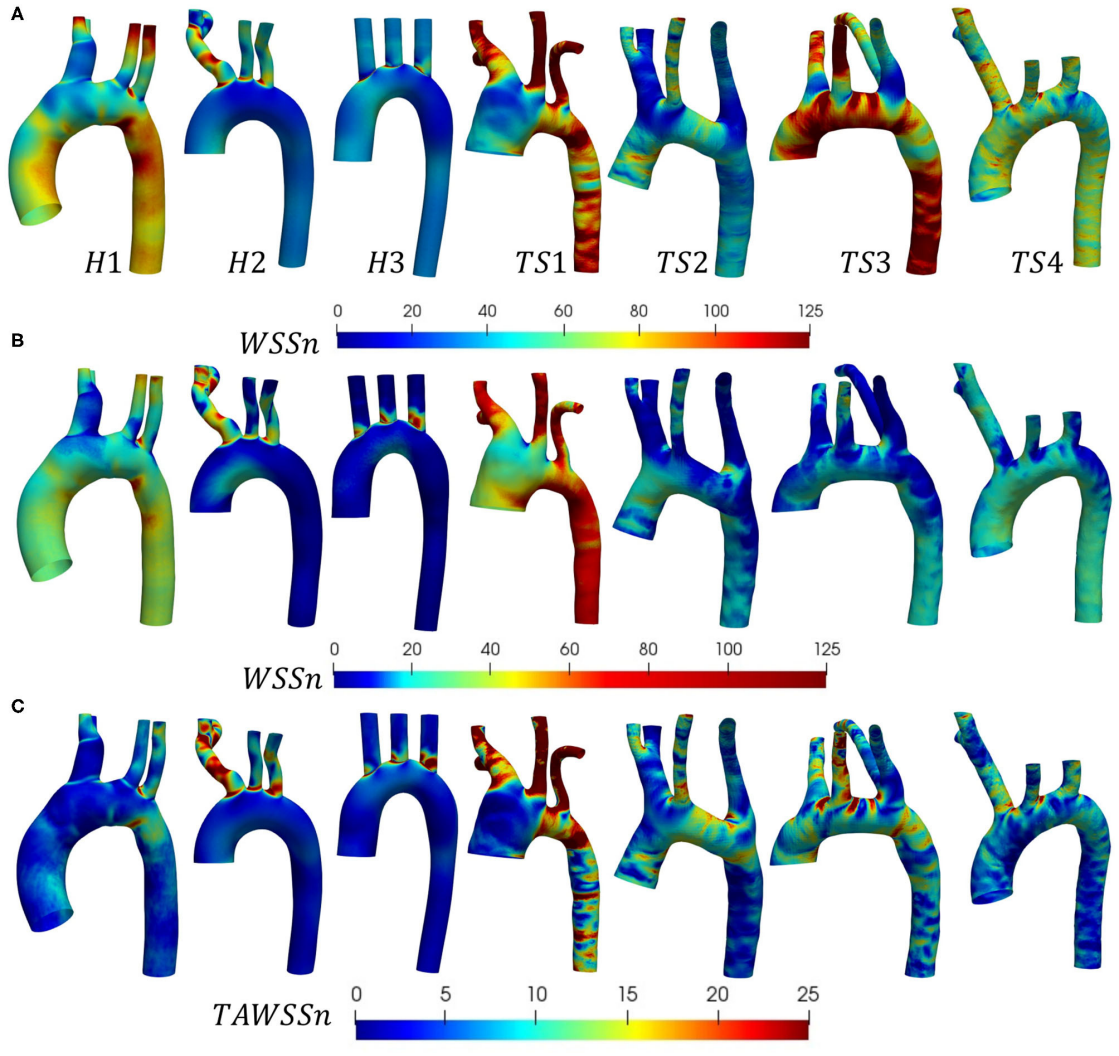
**(A,B)** Instantaneous normalized wall shear stress (WSS_n_), and **(C)** normalized time-averaged wall shear stress (TAWSS_n_) distributions shown (anterior view) for the (H1-H3) healthy, and (TS1-T4) Turner syndrome cases. **(A)** Peak systole and **(B)** maximum deceleration. WSS and TAWSS were normalized with respect to the mean WSS at the inlet for each individual case.

**Figure 7:**
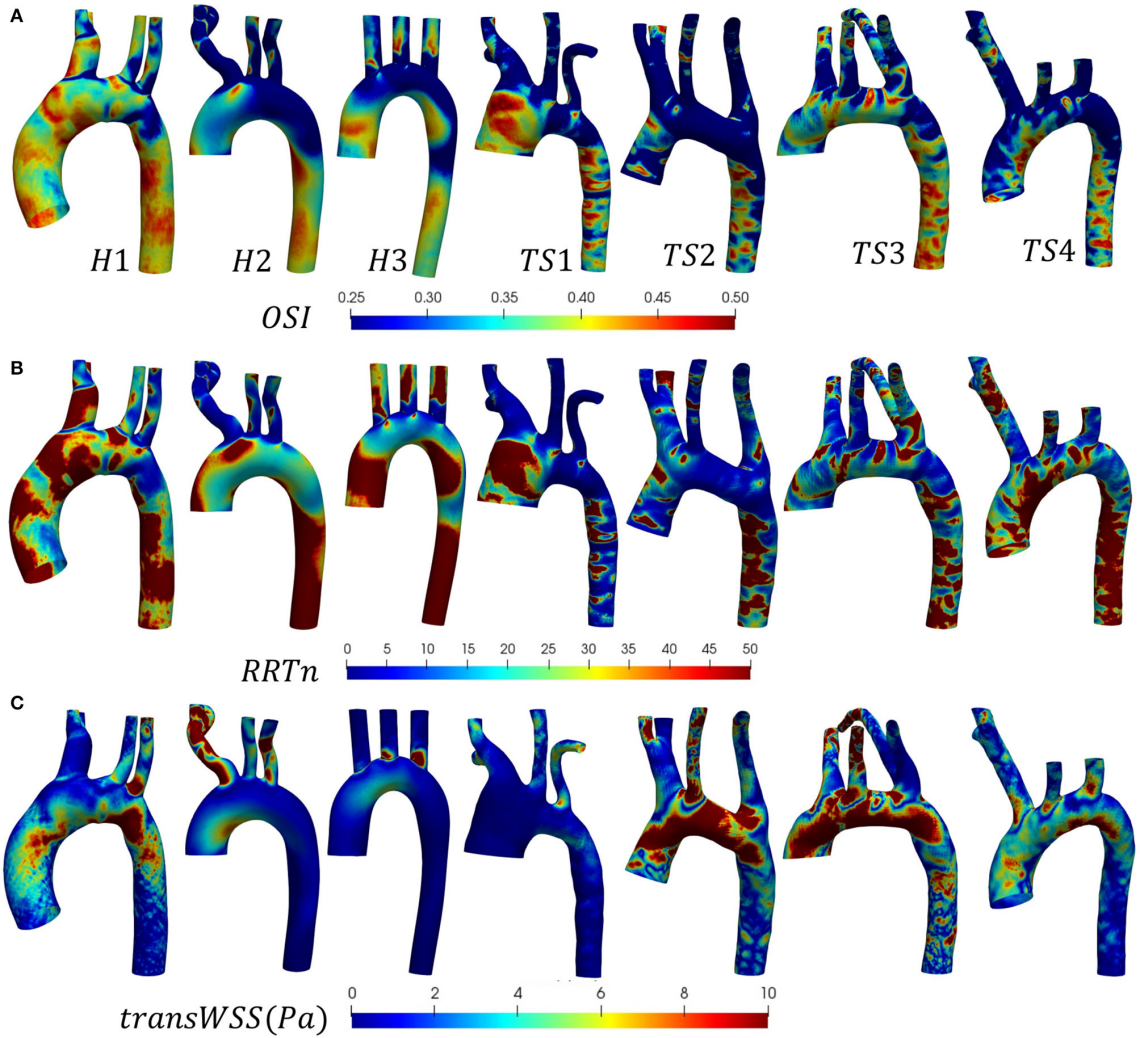
**(A)** Oscillatory shear index (OSI), **(B)** normalized relative residence time (RRTn), based on the surface integral average for each individual case, and **(C)** transverse wall shear stress (transWSS) distributions shown (anterior view) for the (H1-H3) healthy and (TS1-T4) Turner syndrome cases.

**Figure 8:**
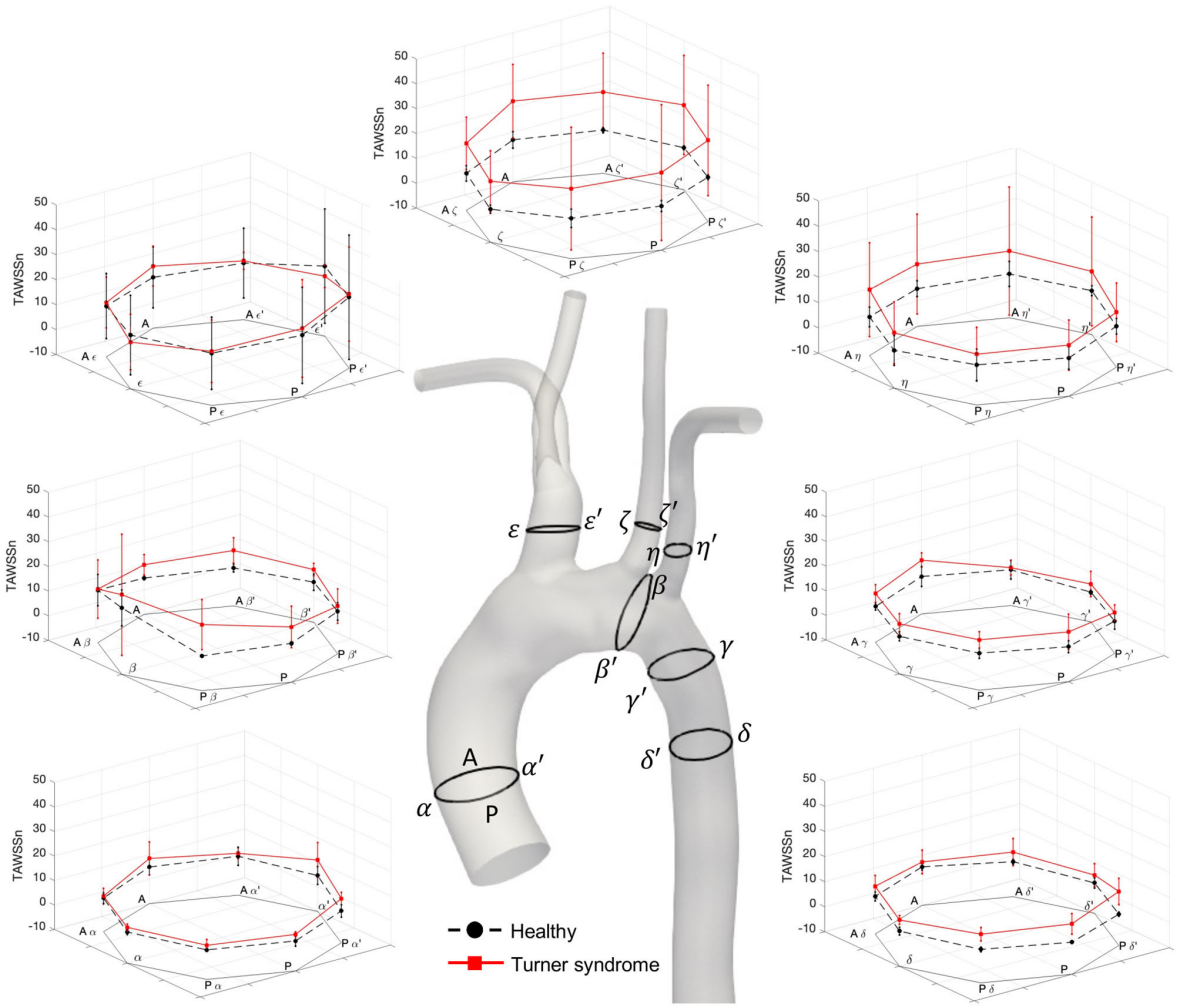
Normalized time-averaged wall shear stress (TAWSS_n_) values at seven cross-sections along the aorta of the healthy (black, dotted lines, as average of H1–H3) and Turner syndrome girls (red, solid lines, as average of TS1–TS4). Cross-sections are located as shown on the 3D model of a healthy (H1) case. Standard deviation shown as error bars at each point.

The distribution of instantaneous normalized wall shear stress (WSSn) at peak systole (t_1_) and maximum deceleration (t_2_) are shown, respectively, in Figures 6A,B. Figure 6C displays the normalized time-averaged wall shear stress (TAWSSn) for all models. Additionally, the oscillatory shear index (OSI), normalized relative residence time (RRTn), and transverse WSS (transWSS) are presented in Figures 7A–C. A further analysis on the regional TAWSSn is provided in Figure 8.

The WSS_n_ distribution differed throughout the cardiac cycle for all models but was most visible at peak systole (Figure 6A) due to the lower values at maximum deceleration (Figure 6B). At peak systole (Figure 6A), each TS aortic geometry presented a highly heterogeneous wall shear stress pattern compared to the less diversified distribution on the aortic wall of the healthy cases. The WSS_n_ pattern at maximum deceleration (Figure 6B) was less clear, with values <20 throughout the majority of the H2, H3, and TS2-TS4 aorta. The peak systolic WSS_n_ had evidently the greatest influence throughout the cardiac cycle, as the TAWSS_n_ (Figure 6C) results were to a large degree similar in character to the WSS_n_ patterns at *t*_1_ (Figure 6A). In the healthy (H1-H3) geometries the majority of the aortic body was exposed to relatively low TAWSS_n_ (0–5) values (Figure 6C), with additional higher TAWSS_n_ regions at the inner ascending aortic wall, and the proximal entrance to the descending aorta in H1. In general for H1–H3, along the greater arch curvature TAWSS_n_ was highest upstream of the branch junctions and lowest downstream. Along the lesser arch curvature, TAWSS_n_ was lowest at the arch entrance and highest at the proximal entrance to the descending aorta. TAWSS_n_ on the three healthy branching arteries was lowest on the proximal walls. In the descending aorta, low TAWSS_n_ was observed on the downstream proximal wall and higher TAWSS_n_ on the distal wall. Of the regions identified on the healthy aortae with the lowest TAWSS_n_ (the inner arch curvature wall, the proximal walls of the three branches, downstream of branch junctions, and the downstream proximal wall of the descending aorta), all were accompanied by elevated OSI and RRTn values (Figures 7A,B). The transWSS (Figure 7C), which provides information on the multi-directionality of WSS, was greater in H1 than H2-H3, with the exception of the brachiocephalic branch in H2. This indicates that the direction of flow in H1 is not parallel to a single axis and experiences fluctuations.

The relatively homogenous distribution of TAWSS_n_ on the healthy aortae was replaced with higher TAWSS_n_ values and highly heterogeneous patterns on the walls of the Turner syndrome geometries (Figure 6C). In the dilated aorta of TS1, low TAWSS_n_ values (0–5) were concentrated at the proximal wall of the ascending aorta, downstream of the three branch junctions, and at the proximal wall of the left subclavian artery. Irregular low shear stresses were also found along the proximal wall of the descending aorta among high shear stress regions. Very high TAWSS_n_ values dominated the three aortic branches and almost circumferentially at two locations along the aorta, the first being between the left common carotid and left subclavian branch, and the second at the transition from the arch to the descending aorta. OSI values were highest at the proximal walls of the ascending and descending aorta, and downstream of the three branch junctions, more specifically at the anterior side. As low magnitude and high oscillatory wall shear stress leads to an increase in the residence time of the blood adjacent to the arterial wall, particularly elevated RRTn values were observed at the ascending and descending aorta, and the distal wall of the brachiocephalic branch for TS1 (Figure 7B). The magnitude of transWSS in TS1 was overall very low, with higher values concentrated to the lesser curvature of the proximal arch and the aortic branches (Figure 7C). Case TS2 was defined by moderate-high TAWSS_n_ and transWSS values (Figures 6C, 7C), extending from the distal wall of the ascending aorta and throughout the majority of the elongated arch. The lowest TAWSS_n_ (Figure 6C), accompanied by high OSI (Figure 7A) and RRTn (Figure 7B), and low transWSS (Figure 7C), was found along the proximal wall of the descending aorta. TS3 had a very heterogeneous time-averaged wall shear stress pattern throughout the entire geometry, with the highest values concentrated to the outer curvature of the arch, near branch junctions, and at the transition from the arch to the descending aorta, as seen in TS1 and TS2. As expected, the OSI and RRTn distributions on the surface of TS3 were also highly heterogeneous, with elevated values dominating the descending aorta (Figures 7A,B). TransWSS (Figure 7C) was highest along the anterior wall of the ascending aorta and arch, as was the case for TS2. TAWSS_n_ heterogeneity in TS4 was concentrated to the outer walls of the ascending aorta, arch, and descending aorta, with low TAWSSn and elevated OSI and RRTn transcending from the lateral wall of the ascending aorta, through the lesser curvature of the arch, to the proximal wall of the descending aorta. The transWSS in TS4 was of lower magnitude than that of TS2–TS3, although the highest values were again found along the anterior wall of the ascending aorta and arch.

A detailed, comparative analysis of the TAWSS and TAWSS_n_ was performed at eight circumferential points of the selected aortic slices for the Turner syndrome group, taken as an average, and compared with those of the healthy group. This investigation revealed distinctly higher TAWSS_n_ values at all eight points of all cross-sections for the TS group (Figure 8). The only regions of the aorta that did not fit this trend were at points ε and ε′ of the brachiocephalic trunk. Cross-sections ζ-ζ′, and η-η′ in the left common carotid and left subclavian branches showed the greatest TAWSS_n_ variation between the healthy and TS groups. When comparing the dimensional TAWSS and non-dimensional TAWSS_n_ values averaged over each analysis plane for both groups (Table 4), the TS group had higher average values at every aortic location for both TAWSS and TAWSS_n_, with the exception of the brachiocephalic branch (ε-ε′) for TAWSS.

**Table 4:** Time-averaged wall shear stress (TAWSS), expressed in Pascals (Pa), and normalized time-averaged wall shear stress (TAWSS_n_) averaged over each analysis plane (α-α′ to η-η') for the healthy (*n* = 3) and the Turner syndrome groups (*n* = 4).

**Analysis plane**	**TAWSS, Pa**		**TAWSS**_********n********_	
	H1–H3^*^	TS1–TS4^*^	H1–H3^*^	TS1–TS4^*^
α-α′	1.04 ± 0.40	1.86 ± 0.63	3.65 ± 1.40	6.55 ± 2.49
β-β′	2.19 ± 1.22	2.86 ± 0.71	7.70 ± 4.33	13.29 ± 4.24
γ-γ′	1.23 ± 0.33	2.08 ± 0.58	4.24 ± 1.11	8.74 ± 2.55
δ-δ′	1.03 ± 0.35	1.94 ± 0.46	3.63 ± 1.18	8.60 ± 1.90
ε-ε′	3.50 ± 0.86	2.52 ± 0.81	13.38 ± 3.32	13.94 ± 3.45
ζ-ζ′	1.79 ± 0.44	3.83 ± 0.98	6.42 ± 1.58	20.36 ± 3.39
η-η′	1.59 ± 0.29	1.97 ± 0.25	5.81 ± 0.99	13.13 ± 2.55

* *Values are given as mean ± standard deviation*.

## Discussion

In our study, patient-specific blood flow simulations were performed through the aortae of four Turner syndrome girls and three healthy girls. In healthy aortae, blood flow patterns range greatly throughout the systolic period (61, 62). The TS girls included in this study had on average larger aortic root diameters (23.66 ± 6.93 mm) and higher peak flow rates (269 ± 49 ml/s, mean ± SD) than the healthy group (19.90 ± 2.48 mm and 241 ± 55 ml/s respectively, mean ± SD). At peak systole, the velocity streamlines revealed the highest flow velocities in the descending aorta of the TS geometries with the largest diameters (TS1 and TS3), but no vortical flow, except at some small recirculation areas in the distal branches of TS3 (Figure 4A). The evolution of helical flow throughout peak to late systole is influenced by the curvature and non-planarity of the arch (63). The visualization of multidirectional blood flow at maximum deceleration (Figure 4B), revealed increased vortical flow in the arch, supra-aortic branches, and descending aorta of the TS patients compared to the healthy controls. This is likely due to the anatomical variants of the TS aortae, rather than the transition from peak systolic flow to diastolic flow, as the peak deceleration value was higher for the healthy group (−13.73 m/s^2^ ± 1.52) than the TS group (−7.98 ± 1.40 m/s^2^, mean ± SD).

Atherosclerosis has a non-uniform distribution within the arterial system and shows a predilection for arterial curvatures and branch junctions. However, it is yet unclear why the endothelium is at times prone or immune to disease, particularly with increasing age. One of our key findings in this study was that compared to the relatively homogeneous distribution of time-averaged wall shear stress on the healthy aortae, there was a highly heterogeneous pattern on the walls of the Turner syndrome geometries. Taking into account the young age of our TS cohort (average age 13 ± 2 years) and their obesity level (75% of the TS girls examined here were obese or overweight, Table 1), which is believed to accelerate the initiation and progression of endothelial dysfunction (64), the higher heterogeneity of WSS distribution found in TS girls may be an indication of atherosclerotic disease manifesting earlier in life in these patients.

Wall shear stress is a biomechanical force, predetermined by fluid flow and arterial geometry, and a key player in the pathogenesis of atherosclerosis (65). Aortic sites where blood flow departs from a laminar, unidirectional pattern, and wall shear stresses are low and/or oscillatory, are often associated with locations of atherosclerosis development (27, 63). However, this link has primarily been established in the literature for adults (27, 63). Studies on other species of younger age (e.g., mice and rabbits) do not necessarily follow the low and/or oscillatory WSS theory (22, 37) and the underlying flow mechanisms related to children are not well-known in general. In our study, there appeared to be a correlation between the presence of aortic abnormalities, as seen in the TS group, and overall elevated TAWSSn. Higher TAWSS values and increased vortical flows may indicate a less atheroprotective environment in the young TS patient cohort studied here, thus not showing complete consistency with the above theory. However, the time-averaged WSS that we used here as part of our conclusions may not be the best indicator of disease predisposition, since atherogenesis may also be associated with the time that endothelial cells are exposed to WSS, rather than TAWSS, during the cardiac cycle (37). Additionally, atherogenesis may not depend only on low and/or oscillatory WSS. Mass transfer of molecules such as Low Density Lipoproteins (LDL) or Nitric Oxide (NO) between the blood and the endothelium may also play a role in the mechanism of atherogenesis, even though they might be related to WSS (37).

### Comparison With Other Studies

As far as we are aware, there are only three published studies on the aortic flow of TS patients. In the 2014 study by Chen et al. (25), a steady flow rate of 4.5l/min was simulated through three patient-specific Turner syndrome aortae (without patient age information), with aortic abnormalities common to two aortic geometries (TS1 and TS2) in our study. Although our study includes more complex simulations, accounting for patient-specific transient flow, similarities can be made with Chen et al. (25) regarding the flow patterns: weak secondary flow in the ascending aorta (Figure 5 slice α-α') became stronger throughout the transverse aorta (Figure 5 slice β-β'); vortical flow was present in the arch of the aortic model with ETA (TS2) (Figure 5 slice β-β'); and the flow in the descending aorta was remarkably different between TS models (Figure 5 slices γ-γ', δ-δ'). While we report a large variation in the flow patterns of the descending aorta among TS1–TS4, we further found much greater asymmetry in the through-plane velocities and stronger secondary flow, especially at maximum deceleration, than Chen et al. (25). In the same study, the authors determined three locations with low WSS (<0.5 Pa): (1) the brachiocephalic artery, (2) the inner side of the aortic arch exit, and (3) the entire inner wall of the descending aorta, which is in agreement with our results of low TAWSS_n_ values, across all healthy and TS aortae.

In a more recent study by Prahl Wittberg et al. (49), a non-patient-specific flow rate of 5L/min was simulated through four different TS geometries using a non-Newtonian flow model (no patient age information provided). The authors found recirculation zones in all TS geometries, specifically at a cross-section in the descending aorta, which was most prominent (in length and width) in the aorta with ETA. This is in good agreement with Figure 5, where in-plane vectors revealed secondary flow in the descending aorta (slice γ-γ') of all TS geometries, as well as H3. While a prominent region of recirculating flow was seen in TS2 with ETA, this region was of a similar length and width to that seen in TS3 and TS4 (Figure 5). In the arch of a TS geometry with ETA, Prahl Wittberg et al. (49) reported a single recirculation zone, as did our results for TS2 at peak systole (*t*_1_) (Figure 5 slice β-β'). At the entrance to the left subclavian branch in the ETA model, Prahl Wittberg et al. (49) visualized recirculating flow, which we also observed anteromedially to the vessel center in TS2 (Figure 5 slice η-η'). The low-velocity recirculatory regions identified by Prahl Wittberg et al. (49) were accompanied by low TAWSS (<0.5 Pa) and high OSI values. Additionally, the authors identified high TAWSS (3 Pa) on the walls of the supra-aortic branches in their normal, dilated, and ETA geometries, and very high TAWSS (5–10 Pa) in the arch of the dilated and ETA models (49). We found similar TAWSS values on the brachiocephalic walls of both the healthy (3.50 ± 0.86 Pa) and TS groups (2.52 ± 0.86 Pa), on the left common carotid walls of the TS group (3.83 ± 0.98 Pa) (Table 4), and on the arch cross-section of TS2 (5.28 ± 1.73 Pa).

The 4D-flow MRI study in the aorta of both healthy and Turner syndrome girls by Arnold et al. (66) found significant differences between the two cohorts (mean age patient group 16 ± 5 years for TS, 17 ± 4 years for healthy controls). Specifically, during early and late systole, and early diastole, helical flow was increased in the ascending and descending regions of TS individuals with significantly larger diameters. Peak systolic velocities did not vary significantly between the TS and control group. In our study, the TS geometries with the largest aortic diameters were TS1 and TS3, for which we demonstrated a similar trend, particularly at mid-diastole (Figure 4C). Additionally, Arnold et al. (66) reported decreased peak systolic WSS throughout the body of the aorta in TS girls, especially at larger diameter regions, when compared to healthy controls. Compared with Figure 6A, we observed a similar pattern on the ascending aortic wall of TS1, but not for TS2-TS4 which had only marginally larger diameters than the healthy controls. Also, our regional TAWSS results (Table 4, Figure 8), averaged over each cross-section, and extended to the supra-aortic branches, concluded that TS girls generally have higher TAWSS and TAWSS_n_ than their healthy counterpart.

### Study Limitations

There are some limitations in this study considering the assumptions made in sections Boundary Conditions and Numerical Method. The assumptions of the arterial wall as rigid and blood as Newtonian are reasonable as the deformation of the arterial wall is relatively small in the proximal aorta and the effects of non-Newtonian blood can be ignored in larger vessels (67). In this study, the applied inlet boundary condition is the volumetric waveform and so information on the spatial profile of the inlet velocity is lost. This was because the 3D velocity profile was not available for the healthy cases. The Murray's law, utilized here to predict the outflow percentages, is based on fully developed laminar Poiseuille flow which is not the case for larger arteries where turbulence occurs (68, 69). However, in the case of absent patient data at the model outlets, and a lack of Windkessel parameter values from the literature, especially for children with Turner syndrome, Murray's law was considered the most appropriate method for the outlet boundary conditions. Furthermore, it was difficult to validate the results of this study since the patient data was obtained retrospectively. Prospective MRI data obtained alongside Doppler ultrasound measurements of blood velocity would allow the validation of the numerical results. Nonetheless, our numerical methodology has been used extensively in the past by our group and other researchers for biomedical engineering problems increasing our confidence in the obtained results (20, 21, 37, 70). Finally, due to the limited availability of retrospective MRI data, especially for children, this study examined only a small cohort of TS patients at a specific age range, which undoubtedly limits the statistical significance of our results.

### Clinical Significance

Computational fluid dynamics can be a very powerful tool in cardiovascular medicine. The evaluation of hemodynamic parameters of patient-specific aortic models that are challenging to measure *in vivo* can improve our understanding of cardiovascular disease processes, thus enhancing diagnostic capabilities, and progressing toward patient-specific precision medicine. TS girls and women face a lifelong battle with a broad spectrum of cardiovascular concerns, from congenital heart abnormalities to an increased risk of hypertension, ultimately reducing life expectancy. The current management of cardiovascular conditions in TS is the same as in the general population, due to a lack of understanding of the developmental origins of the cardiovascular manifestations seen specifically in TS (4). The key clinical question is whether any of the cardiovascular risk in TS patients is modifiable. Currently, known modifiable risk factors are hypertension and obesity and therefore the treatment of hypertension and avoidance of being overweight and obese is necessary for this group of patients. This begs the question - would this alter the flow dynamics more favorably too? In this study, the anatomical abnormalities observed in the aortae of young TS girls, the majority (75%) of which were obese or overweight, were accompanied by abnormal flow patterns and highly non-uniform distribution of wall shear stresses, which may promote the development of cardiovascular diseases earlier in life. The type of analysis presented in this study could be used clinically to predict patients at higher risk and therefore be more pro-active in lifestyle measures. Aortic dilatation, although rare, is more common in TS patients and is catastrophic, associated with high mortality. Other risk factors are bicuspid aortic valves, hypertension and previous aortic surgeries, as well as pregnancy. It is still very difficult to predict even if aortic size index, a criterion to estimate the probability for aortic dissection in TS patients, has been evaluated correctly (71). Pregnancy in some recommendations is contraindicated in all of TS due to this risk. CFD modeling could help in this direction (72). Considering the excess of morbidity and mortality, the early diagnosis of cardiovascular changes associated with Turner syndrome is essential, and given the advantages of CFD in monitoring these changes, this method should be used alongside the standard Doppler echocardiography and magnetic resonance imaging in the clinical assessment of these patients.

## Conclusion

Personalized flow in seven patient-specific aortic geometries (four TS, three healthy) was investigated in a comprehensive investigation of Turner syndrome children, using computational fluid dynamic methods. The visualization of multidirectional blood flow revealed increased vortical flow in the arch, supra-aortic branches, and descending aorta of the TS girls compared to the healthy controls, but no significant difference was found in the ascending aorta. This study found that young Turner syndrome patients, at about 13 years of age, exhibit markedly elevated TAWSS values when compared to age-and gender-matched healthy controls, suggesting a correlation with the presence of aortic abnormalities. The Turner syndrome girls, 75% of whom were obese or overweight, also display a highly heterogeneous pattern of TAWSS on the aorta, compared to the relatively homogeneous distribution of the healthy aortae, which may be an indication of atherosclerotic disease manifesting earlier in life in these patients. These results are not fully consistent with the low and/or oscillatory WSS theory of atherogenesis, which nonetheless may depend also on other factors, e.g., age, endothelial cells' exposure time to WSS and not TAWSS, and mass transfer of LDL and NO molecules to the endothelium, as explained here. For TS children, age, obesity and aortic morphology may, therefore, play a key role in assessing cardiovascular risk. Further investigations are required on the role of mechanobiological factors on atherogenesis in healthy and diseased children, and the hemodynamics in different age groups of TS patients in particular.

## Data Availability Statement

The original contributions presented in the study are included in the article/supplementary material, further inquiries can be directed to the corresponding author.

## Ethics Statement

The studies involving human participants were reviewed and approved by UK Health Research Authority, London–Westminster Research Ethics Committee (IRAS Project ID: 252866, REC Reference: 18/LO/2052). Written informed consent to participate in this study was provided by the participants' legal guardian/next of kin.

## Author Contributions

All roles according to CRediT (contributor roles taxonomy). LJ: methodology, software, formal analysis, writing—original draft, and visualization. AK and LJ: conceptualization, investigation, data curation, writing—review and editing, and project administration. AK, AM, RA, and PH: resources and writing—review and editing. AK: supervision and funding acquisition. All authors contributed to the article and approved the submitted version.

## Conflict of Interest

The authors declare that the research was conducted in the absence of any commercial or financial relationships that could be construed as a potential conflict of interest.

## Abbreviations

TS: Turner syndrome
ETA: Elongated transverse arch
BAV: Bicuspid aortic valve
CoA: Coarctation of the aorta
BCA: Brachiocephalic artery
RSA: Right subclavian artery
RCCA: Right common carotid artery
LCCA: Left common carotid artery
LSA: Left subclavian artery
WSS: Wall shear stress
TAWSS: Time-averaged wall shear stress
transWSS: transverse wall shear stress
OSI: Oscillatory shear index
RRT: Relative residence time.
